# Spontaneous organ-specific eye autoimmune disease resulting from two engineered gene mutations compromising immune tolerance

**DOI:** 10.3389/fimmu.2026.1923275

**Published:** 2026-09-22

**Authors:** Mianmian Yin, Mark S. Anderson, Clifford A. Lowell, Anthony L. DeFranco

**Affiliations:** 1 Department of Microbiology and Immunology, University of California, San Francisco, San Francisco, CA, United States; 2 Diabetes Center, University of California, San Francisco, San Francisco, CA, United States; 3 Department of Medicine, UCSF, San Francisco, CA, United States; 4 Department of Laboratory Medicine, University of California, San Francisco, San Francisco, CA, United States

**Keywords:** AIRE, autoimmunity, *Lyn*, T cell, TCR, transgenic, uveitis

## Abstract

In order to address the question of how genetic compromises in multiple immune tolerance mechanisms might confer susceptibility to autoimmune disease development, we have bred together a partial loss of function in Aire, which is important for central tolerance of T cells, with a deficiency of the intracellular protein tyrosine kinase Lyn, which is important for function of inhibitory receptors of dendritic cells, B lymphocytes, and myeloid cells. These *Aire^GW/+^Lyn^-/-^* mice spontaneously develop severe eye autoimmunity, characterized by retinal destruction and damage to the surrounding uvea, referred to as retinitis or uveoretinitis. Remarkably, this disease develops between 6 and 9 weeks of age in 50% of the mice, whereas the remaining 50% never develop disease. This disease is completely dependent on interphotoreceptor retinoid-binding protein (IRBP), a protein expressed primarily in retinal photoreceptor cells and, in the thymus under the control of Aire. Moreover, the dominant T cell epitope of this protein has been identified, and the corresponding autoreactive T cells can be characterized using peptide/MHC tetramers and flow cytometry. In this review, we summarize findings to date with this recently developed mouse model of spontaneously developing organ-specific autoimmune disease. In addition, we discuss conclusions from these findings that provide mechanistic insights into disease initiation, discuss similarities and differences to other mouse models of uveoretinitis, and discuss the complementary nature of what likely can be learned with this model compared to the highly studied NOD mouse model of autoimmune diabetes.

## Introduction

Autoimmune diseases commonly exhibit strong heritability but incomplete penetrance. Studies of identical twins consistently illustrate this paradox: genetic identity substantially increases risk of disease, yet many twin pairs remain discordant (1), suggesting that stochastic and environmental factors, such as infections, shape whether susceptibility becomes disease. Genome-wide association studies (GWAS) demonstrate that in most cases, human autoimmunity involves polygenic susceptibility, with any individual genetic variant contributing a small degree of risk (1, 2). Many loci map to pathways controlling antigen presentation, lymphocyte activation thresholds, and immune regulation. Notably, there is frequent overlap of susceptibility loci across clinically distinct autoimmune diseases, consistent with shared “core” tolerance and activation mechanisms that can manifest in different target organs depending on additional modifiers.

Rare monogenic autoimmune syndromes demonstrate what happens when key tolerance checkpoints fail completely. Loss-of-function mutations in FOXP3 cause severe systemic autoimmunity due to defective regulatory T cells (Tregs) in humans (IPEX syndrome: immunodysregulation, polyendocrinopathy, enteropathy, X-linked syndrome) and in mice (scurfy mice) (3–5). Similarly, individuals with autoimmune polyendocrine syndrome type 1 (APS-1) were found to have inherited mutations in AIRE (6–8). Generation of corresponding *Aire* mutations in mice similarly resulted in multi-organ autoimmunity, which has been shown to result from loss of expression of tissue-specific autoantigens (TSAs) in thymic medullary epithelial cells, expression that is important for T cell central tolerance to such proteins (9). In addition to autosomal recessive AIRE deficiency, AIRE variants in one copy, together with a functional AIRE allele, have been found in human families with dominantly inherited autoimmune disease syndromes (8). Such alleles encode a defective Aire protein that interferes with the function of the normal Aire protein in the cell, resulting in greatly reduced expression of TSAs in the thymus (10).

In addition to confirming the central importance of the FOXP3 and AIRE genes for immune tolerance, mouse models of autoimmune diseases have been highly informative about immune mechanisms of disease in many ways. These models include both immunization-induced models of autoimmunity, such as experimental allergic encephalomyelitis (EAE), and spontaneous models of lupus (2, 11) and of autoimmune diabetes in the NOD mouse model (12–14). More recently, genetically engineered mice with autoimmune phenotypes have been reported and studied. To address how multiple genetic susceptibility factors, each with modest individual effects, but acting on distinct immune tolerance checkpoints, might interact to compromise immune tolerance and lead to organ-specific autoimmunity, we bred a novel mouse model combining two genetically engineered mutations. For this purpose, we chose a dominant-negative *Aire* variant (G228W; termed *Aire^GW^*), originally identified in an Italian family with dominantly inherited autoimmunity, and introduced it into the murine *Aire* locus. In mice with one *Aire^GW^* allele and one wild type *Aire* allele, there was a considerable but not complete loss of Aire function as assessed by TSA expression in the thymus (10). On the C57BL/6 background, *Aire^GW/+^* caused only mild inflammation of the lacrimal and salivary glands, whereas in NOD mice it triggered much more severe autoimmunity in multiple organs (10). The second mutation we chose was deletion of *Lyn*, which is an intracellular protein tyrosine kinase that is important for the function of many inhibitory receptors in dendritic cells and B lymphocytes. C57BL/6 *Lyn^-/-^* mice spontaneously develop systemic lupus erythematosus (SLE), starting at about 3 months of age (15, 16), and its deletion in dendritic cells or in B cells is sufficient to result in SLE, indicating that it plays a role in immune tolerance in both cell types (16, 17). *Lyn^-/-^* mice do not exhibit organ-specific autoimmunity, although *Lyn* deletion in dendritic cells leads to increased barrier inflammation (17). Remarkably, 50% of the mice with the *Aire^GW^* and *Lyn^-/-^* mutations on an autoimmune resistant genetic background (C57BL/6) were found to develop severe spontaneous autoimmune eye disease involving destruction of the retina and inflammation of the surrounding uvea (18), referred to here as uveoretinitis. In this review, we summarize key observations related to this eye autoimmune disease in *Aire^GW/+^Lyn^-/-^* mice and the main conclusions derived to date from this model. In addition, the insights derived from this mouse model of spontaneous autoimmune disease are discussed in relation to what is known about the mechanisms of experimentally induced uveoretinitis models in mice targeting the same autoantigen or retina-expressed neo-self antigens, as well as to mechanisms of spontaneous autoimmune diabetes in NOD mice.

## Organ involvement and temporal progression of uveoretinitis in *Aire^GW/+^Lyn^-/-^* mice

Histological examination of *Aire^GW/+^Lyn^-/-^* double mutant mice demonstrated remarkably selective organ involvement. Lacrimal and salivary glands exhibited lymphocytic infiltrates comparable to *Aire^GW^*^/+^ single mutants, while the stomach, pancreas, liver, lungs, and sciatic nerve showed minimal to no pathology. Anti-dsDNA IgG titers remained unchanged between double mutants and *Lyn*^-/-^ mice, indicating that reduced Aire function did not modify lupus-like manifestations. The eye emerged as the organ uniquely affected by a synergistic interaction between the two engineered mutations, with severe retinal lymphocytic infiltration in 48 of 100 double mutants but complete absence in *Lyn^-/-^* controls and 3% incidence in *Aire^GW/+^* mice (18). Longitudinal funduscopy and retinal histology revealed a stereotypical disease progression beginning around 6 weeks of age, occasionally as early as 5 weeks. Initial manifestations included retinal vessel swelling, perivascular exudates, mononuclear cell accumulation around vasculature and in the subretinal space, and fluorescein-confirmed vascular leakage (18). By approximately 2 months of age, inflammation intensified with extensive mononuclear infiltration into the choroid and subretinal regions, destroying the rod outer segment layer. The spread of inflammation beyond the retina into the surrounding uvea has led us to describe the inflammation as uveoretinitis. Terminal stages featured complete architectural destruction, scarring, and atrophy. This temporal pattern mirrored that of *Aire^-/-^* mice and proved highly reproducible across cohorts within a single specific-pathogen free animal care facility (19). In all cases, if one eye exhibited inflammation, the other eye was similarly affected. Disease incidence was also indistinguishable between males and females. Critically, mice without uveoretinitis by 8–10 weeks never developed disease during extended observation, identifying a definitive window for disease susceptibility (18).

## Specificity of the autoimmune response in *Aire^GW/+^Lyn^-/-^* mice

Initial studies demonstrated that approximately 50% of *Aire^GW^*^/+^*Lyn^-/-^* mice develop uveoretinitis, with disease strongly correlating with CD4^+^ T cell responses against a rod cell cytoplasmic protein, interphotoreceptor retinoid–binding protein (IRBP), a protein that is expressed primarily in photosensing neurons, and also in the thymus under the control of Aire (18). IRBP had previously been shown to be the target of eye autoimmunity in *Aire^-/-^* mice. Moreover, the pathogenic potential of an autoimmune response to IRBP has been demonstrated in the immunization-induced autoimmunity model experimental autoimmune uveitis (EAU) (19). Additionally, autoimmune responses to IRBP have been detected in human uveitis patients, suggesting potential relevance to human disease (20). To determine whether IRBP is indispensable for ocular autoimmunity in the double mutant mice, *Aire^GW/+^Lyn^-/-^IRBP^-/-^* triple-mutant mice were generated and monitored by funduscopy through 3 months of age. While control mice retaining one or both IRBP alleles (*Aire^GW/+^Lyn^-/-^IRBP^+/-^* and *Aire^GW/+^Lyn^-/-^IRBP^+/+^*) exhibited the expected ~50% disease penetrance, IRBP-deficient animals were completely protected from uveoretinitis. This absolute requirement for IRBP expression establishes it as the critical target autoantigen driving disease rather than a target of epitope spreading from some other initiating self antigen (21).

Among three known IRBP epitopes in C57BL/6 mice (amino acids 271-290, called P2, amino acids 651-670, called P6, and amino acids 771–790 called P7), CD4 T cells recognizing the P2 epitope, as assessed using class II MHC (I-A^b^) tetramers, were more abundant than CD4 T cells recognizing the other two epitopes (18). For this reason, most attention has been focused on P2-specific CD4 T cells in the *Aire^GW/+^Lyn^-/-^* mice. Single-cell TCR sequencing of magnetically enriched, sorted P2 tetramer-binding CD4^+^ T cells from pooled eye-draining lymph nodes revealed a remarkable diversity of TCRs recognizing the P2/I-A^b^ epitope in mice with or without autoimmune eye disease, and greater expansion of predominant clones in mice with disease (21). The IRBP specificity of the top five expanded TCR clonotypes in mice with uveoretinitis was assessed by retroviral transduction into a TCR-deficient 58α^-^β^-^ T cell hybridoma cell line carrying an NFAT-GFP reporter. The five TCRs tested were all specific for the IRBP P2 epitope, but with a range of sensitivities, and did not cross-react with the P6 or P7 epitopes. Collectively, these findings establish that in mice with defective Aire function, the IRBP P2-specific repertoire is diverse and that uveoretinitis associates with the selective expansion of CD4 T cell clones capable of recognizing the P2 epitope naturally processed from IRBP (18, 21).

## IRBP P2-specific TCR transgenic mice exhibit spontaneous uveoretinitis

To facilitate mechanistic investigation of the immunologic characteristics of IRBP P2-specific CD4^+^ T cells, transgenic mice were created that express a P2-specific TCR clonotype with intermediate sensitivity to P2 (21). Constructs encoding the TCR α and β chains under the control of T cell-restricted regulatory elements were injected into mouse zygotes to generate TCR transgenic mice, referred to as P2.U2^+/-^ transgenic mice. While the development of T cells in these transgenic mice proceeded to the single-positive stages, both CD4^+^ and CD8^+^ single-positive (SP) populations were reduced in the thymus of P2.U2^+/-^ mice compared to non-transgenic mice, and only 1% of the CD4^+^ SP thymocytes bound to the P2 tetramer. This low percentage probably reflects less than optimal expression of one or both of the transgenes combined with negative selection due to recognition of the autoantigen, as discussed below. In any case, 82% of P2.U2^+/-^ mice on an otherwise wild-type C57BL/6 background lacking Aire or Lyn mutations spontaneously developed uveoretinitis by 5–7 weeks of age when analyzed. These results demonstrate the pathogenic potential of CD4 T cells recognizing the P2 epitope of IRBP.

Earlier studies had also demonstrated the pathogenic potential of IRBP specific CD4 T cells for causing autoimmune uveoretinitis in mice. Horai, Caspi and colleagues used the B10.RIII strain of mice, which are highly susceptible to IRBP immunization-induced uveoretinitis. They created several different lines of transgenic mice in the B10.RIII genetic background that express a TCR, called R161, that recognizes a relevant epitope from IRBP in this strain (22). Transgenic mouse lines expressing high or moderate levels of the R161 TCR (called R161H and R161M, respectively) spontaneously developed uveoretinitis, with disease being more severe in the mice with higher expression of the transgenic TCR (22). Both the P2.U2 and the R161 models underscore IRBP’s centrality as an autoantigen capable of driving ocular autoimmunity.

## Impaired thymic negative selection of P2-specific T cells in Aire-deficient mice

Previous studies had indicated that Aire-regulated expression of IRBP in the thymus could remove P2-specific CD4 T cells from the repertoire, suggesting negative selection (10). In the P2.U2^+/-^ transgenic mice, only 1% of the CD4^+^ SP thymocytes bound to the I-A^b^/P2 tetramer, a low percentage that suggested the possibility of strong negative selection. In agreement with this hypothesis, intracellular staining for cleaved caspase 3, a manifestation of apoptosis, showed an approximately two-fold increase in the fraction of positively staining transgenic CD4^+^ SP thymocytes compared to non-transgenic CD4^+^ SP thymocytes (21). Moreover, there was a substantial number of CD4^+^ T cells in the periphery, but these T cells were almost totally unresponsive to IRBP P2 stimulation *in vitro*, suggesting that they may have been primarily cells expressing TCRs other than the transgenic TCR.

To test whether the apoptosis observed in thymocytes from P2.U2^+/-^ TCR transgenic mice was dependent on IRBP expression in the thymus, we bred the P2U2 transgenic mice to mice with mutations in *IRBP* or *Aire*. While *IRBP^-/-^* mice completely lack IRBP expression in the eye and in the thymus, *Aire^GW/+^* mice retain normal IRBP expression in the retina together with ~10-fold reduced expression in the thymus. Flow cytometric analysis of the thymocytes from these mice revealed that P2.U2^+/-^*Aire^GW/+^* and P2.U2^+/-^ *IRBP^-/-^*mice displayed reductions in the frequencies of CD4^+^SP apoptotic cells, compared to that seen in the P2.U2^+/-^ mice. Thus, the increased incidence of apoptosis of CD4^+^ SP thymocytes seen in the P2.U2^+/-^ mice was due to IRBP expression in the thymus, leading to presentation to the transgenic thymocytes and negative selection. Correspondingly, ~12% of the CD4^+^ SP thymocytes from P2.U2^+/-^ *IRBP^-/-^*mice bound to the P2 tetramer, a large increase compared to the ~1% tetramer-binding CD4^+^ SP thymocytes observed in P2.U2^+/-^ mice. Moreover, the *Aire^GW/+^* mutation phenocopied IRBP deficiency in permitting escape from negative selection of the P2.U2 TCR transgenic thymocytes (21). Thus, negative selection of P2.U2 transgenic CD4^+^ SP thymocytes was prominent and depended on Aire-regulated IRBP expression in the thymus. As would be expected from the effects of these mutations on negative selection in the thymus, the peripheral CD4^+^ T cells in the P2.U2^+/-^*Aire^GW/+^* and P2.U2^+/-^ *IRBP^-/-^* mice had elevated fractions of cells that bound P2-tetramer and, moreover, these cells demonstrated robust *in vitro* responsiveness to P2 peptide through CD69 upregulation and proliferation—responses virtually absent in P2.U2^+/-^ mice with intact Aire. Notably, some peripheral P2-specific CD4^+^ T cells from Aire-sufficient transgenic mice exhibited high CD44 expression *ex vivo*, suggesting *in vivo* encounter with IRBP, since elevated expression of this activation marker was absent in T cells from IRBP-deficient mice. Another possible explanation for this observed CD44 expression is that it could result from homeostatic proliferation seen in the context of lymphopenia, although the P2.U2^+/-^ are not clearly T cell lymphopenic. The presence or absence of lymphopenia may be a significant parameter for mouse models of autoimmunity, as is discussed further below.

All P2.U2^+/−^*Aire^GW/+^* mice rapidly developed uveoretinitis by 5–7 weeks of age, whereas P2.U2^+/−^*IRBP^−/−^* animals remained disease-free despite equivalent thymic selection defects, indicating that spontaneous uveoretinitis in these mice requires IRBP expression in the eye, as is the case for *Aire^GW/+^Lyn^-/-^* mice. As mentioned above, the majority but not all P2.U2^+/-^ *IRBP^+/+-^* mice spontaneously developed uveoretinitis, so disease induction by P2.U2 transgenic T cells appears to be enhanced by decreased Aire function. In any case, in the context of a high IRBP P2-specific CD4 T cell precursor frequency resulting from expression of the P2.U2 transgenes, central tolerance mediated by Aire and normal expression of Lyn are insufficient to prevent autoreactivity in most individual mice. Nonetheless, the P2.U2 transgenic mice represent an excellent new model to study in detail how IRBP-specific CD4 T cells that escape central tolerance due to genetic compromise of Aire function can be activated in the periphery or silenced by peripheral tolerance mechanisms.

## TCR transgenic models of uveoretinitis in mice

In addition to the two IRBP-specific TCR transgenic mice mentioned above (P2.U2 and R161), both of which spontaneously develop uveoretinitis, there have been studies of two other TCR transgenic models employing neo-self antigens with transgene-directed expression in the retina (23, 24). In one model, the model antigen hen egg lysozyme (HEL) was expressed in the retina using IRBP regulatory regions and then combined with a HEL-specific transgenic TCR (3A9) expressed in CD4 T cells. In this model, the model neo-self antigen is expressed both in the retina and the thymus, the latter under the control of Aire. The resulting double transgenic mice all developed severe uveoretinitis starting at the time of eye opening in young mice (day 20-21) (23). These mice exhibited considerable T cell lymphopenia, which is thought to be due to a combination of strong negative selection in the thymus and strong transgene expression in thymocytes (hence, these mice presumably have reduced numbers of thymocytes expressing endogenous TCRs).

Adoptive transfer experiments in this model have provided additional insight into the mechanism by which autoimmune inflammation is initiated (25). Transfer of naive HEL-specific T cells into the HEL-IRBP single transgenic mice resulted in weak proliferation of some of the transferred CD4 T cells, and no uveoretinitis. Uveoretinitis could be induced in the adoptively transferred mice if they were infected with a virus (murine cytomegalovirus, MCMV) engineered to encode the HEL epitope, but not if they were infected with the virus lacking the HEL epitope. Thus, in this circumstance, the autoimmune response to the neo-self-antigen-expressing retina required molecular mimicry with an infectious agent capable of stimulating a vigorous immune response of the self-reactive CD4 T cells (26). In addition, two different HEL-IRBP transgenic lines with different levels of expression of HEL were tested, and more severe autoimmune damage was induced in the mice with higher expression of the neo-self antigen. As is discussed below, studies with the IRBP-specific R161 TCR transgenic mice have also demonstrated that molecular mimicry, in that case with gut microbes, appears similarly to be necessary to induce spontaneous autoimmune disease (27). These results raise the question of why the double transgenic HEL/anti-HEL mice develop autoimmunity. One contributing factor is likely to be the lymphopenia seen in these double transgenic mice, but some second factor is likely necessary. As the immune attack in these mice occurs at the time of eye opening in the mice, this may contribute, perhaps by light damage inducing some eye inflammation or release of HEL from the eye to the draining lymph nodes.

A second neo-self antigen mouse model of uveoretinitis has also been created and studied (24), and its characteristics are distinctive. In this system, the neo-self antigen is *E.coli* β-galactosidase, expressed from the retina-specific promoter for arrestin (also called retinal S-antigen), a signaling protein of light-sensing neurons, and it was combined with either of two transgenic TCRs that recognize epitopes from this protein in either B10.A mice (original studies) or in C57BL/6 mice (more recent studies; BG2 TCR). In this double transgenic system with either TCR and corresponding genetic background, spontaneous uveoretinitis is not observed, despite absence of detectable expression of β-galactosidase in the thymus and an apparent lack of negative selection of the transgenic thymocytes. The reason why this TCR transgenic system does not spontaneously develop uveoretinitis, in contrast to the other systems described above, is not entirely clear, but nonetheless, this system has provided a number of interesting insights.

In addition to the lack of uveoretinitis in the β-galactosidase double transgenic mice, these mice are resistant to EAU induction with an immunization protocol that induces uveitis in the neo-self antigen single transgenic mice, indicating an active tolerance process (24). Uveoretinitis could be induced by an adoptive transfer of the B10.A TCR transgenic T cells into the antigen-transgenic mice, but only in the context of a recipient lacking T cells (lymphopenic) and with depletion of CD25^+^ cells from the transferred cells. These results suggest that Treg are likely a major mechanism of peripheral tolerance to this neo-self antigen with these TCR transgenic T cells. Subsequent studies with BG2 TCR transgenic T cells found that Treg fate for the TCR transgenic CD4 T cells could be acquired by exposure to β-galactosidase injected into the eye and this would prevent disease in immunization-induced uveitis (28). Moreover, depletion of Treg selectively in the eye increased the incidence of EAU upon immunization, and now resulted in a minority of the double transgenic mice developing spontaneous eye disease (29). Thus, Treg appear to be an important component of the observed tolerance to β-galactosidase of these TCR transgenic + neo-self-antigen transgenic mice. These results are in agreement with a variety of studies demonstrating that eye tissue has immunosuppressive properties that favor Treg development (30). Similarly, naive CD4 T cells expressing the R161 transgene are induced to become Treg by injection into the eye, which expresses endogenous IRBP at substantial levels (31). Thus, the insights derived from this experimental model appear to have some general applicability.

## Limited thymic niche for Treg fate determination of P2-specific thymocytes

It is now well established that Aire promotes central tolerance to a substantial number of TSAs by two mechanisms: clonal deletion and commitment to the FoxP3^+^ Treg differentiation state in the thymus, but what controls the balance between these two fates is incompletely understood (9). TCR avidity for peptide/MHC and/or expression level and presentation efficiency of the self antigen may be among the relevant parameters. To address the propensity of thymocytes expressing the P2.U2 transgene to adopt the Treg cell fate and escape negative selection, the P2.U2 transgenic mice were bred to a FoxP3-green fluorescent protein (GFP) reporter. Remarkably, thymic Treg commitment among P2-tetramer^+^ cells appeared to be minimal (less than 1%) (21). This latter observation might reflect a small niche for Treg fate determination, as indicated by experiments with TCR transgenic models specific for the prostate antigen Tcaf3 (32). To test this possibility, mixed bone marrow chimeric mice were generated in which various ratios of bone marrow cells from P2.U2^+/-^ transgenic mice and from wild-type mice were used to reconstitute lethally irradiated recipient mice. Interestingly, the frequency of CD4^+^ SP thymocytes expressing the Treg-specific transcription factor FoxP3 was inversely correlated with P2.U2^+^ precursor frequency and also depended critically on Aire function (21). Therefore, Treg differentiation of P2.U2 TCR transgenic thymocytes was dependent on expression of an Aire-dependent gene, presumably IRBP, in the thymus and exhibited a limited niche, as previously observed for the prostate-specific antigen Tcaf3, the expression in the thymus of which is also Aire-dependent.

As was seen for P2.U2 thymocytes, only a small fraction of R161H and R161M thymic TCR^+^ cells expressed FoxP3 (22), which is consistent with the observation that there is a small niche for IRBP-specific Treg fate determination that is limiting in the context of the high frequency of IRBP-specific thymocytes in these TCR transgenic mice. Whether or not R161H and R161M transgenic thymocytes were subject to negative selection due to IRBP expression in the thymus of B10.RIII mice was not directly analyzed.

It should also be noted that the fraction of P2-specific P2.U2 CD4 SP thymocytes that expressed FoxP3 was always less than 20%, suggesting that negative selection is the more robust effect of IRBP P2-presentation to the transgenic thymocytes and that Treg differentiation is a less prevalent outcome. Because of this limited niche in the thymus for Treg differentiation, in the context of the transgenic P2.U2^+^ mice with a high precursor frequency of IRBP P2-specific thymocytes, negative selection was the predominant mechanism of central tolerance and Treg differentiation was observed but appeared to contribute to tolerance to a lesser degree. In most of the mice, the resulting Treg were unable to prevent spontaneous development of autoimmune tissue destruction caused by the relatively few transgenic T cells that escaped both negative selection and Treg differentiation (21).

Although defects in Aire function compromised the differentiation of P2 IRBP-specific T cells into Treg, clearly it is possible for autoreactive CD4 T cells to be induced to become Treg in the periphery, and indeed the eye is especially able to promote Treg differentiation. For example, in the R161 transgenic system, it was found that injection of naive R161H T cells into the eye induced their differentiation into Treg (31)and also into anergic T cells, as determined by single cell transcriptomic analysis (33). Interestingly, if the R161H T cells were first primed to IRBP *in vivo*, then injected into the eye, they did not convert to Treg, demonstrating a limitation in the ability of the eye microenvironment to induce differentiation into Treg.

## Role of the microbiota in mouse models of uveoretinitis

A popular hypothesis is that spontaneous autoimmunity can be triggered by an infection, either through innate immune stimulation that enhances the autoimmune reaction by an adjuvant-like effect, or by molecular mimicry (34–36). In molecular mimicry, some of the T cells responding to an infectious agent cross-react with a critical self-antigen such that after the T cells become activated by reaction against the infectious agent; subsequently they can initiate the autoimmune attack on the affected organ (35). In support of one or another of these possibilities, microbiota have been found to be important for a number of experimental autoimmune diseases in mice (34, 36). With regard to uveoretinitis, in the spontaneous eye autoimmunity seen in transgenic mice expressing the R161 IRBP-specific TCR, depletion of microbiota by treatment with a cocktail of antibiotics prevented the development of disease (27). In addition, the transgenic TCR-expressing T cells exhibited activated effector phenotypes in the gut lamina propria of conventionally housed mice, where exposure to gut-derived antigens may occur. Thus, molecular mimicry is an attractive hypothesis for how the R161 transgenic mice develop eye autoimmunity. In addition, it may be that priming in the periphery (for example, in the gut lamina propria or mesenteric lymph node) of the R161 TCR transgenic T cells is required for them to be able to cross the blood-retina barrier and access uninflammed retinal tissue and initiate disease (36). Remarkably, EAU induced by immunization of C57BL/6 mice with a peptide representing a T cell epitope from IRBP also is attenuated by antibiotic treatment 1 week prior to immunization, suggesting a contributing role for the microbiota in this version of EAU (37). In addition, studies with the HEL neo-self antigen double transgenic system described above have observed that the HEL-specific transgenic T cells can become activated to HEL peptide/MHC complexes presented by antigen-presenting cells in the gut and have proposed that T cells activated in this location may contribute to the strength of the autoimmune response (38). These findings apparently are not related to a cross-reaction with antigens of the gut microbiome and instead may reflect an adjuvant-like property of the gut microbiome. Evidence suggesting that the microbiome may contribute to human uveitis has recently been reviewed (39).

So far, a role for the microbiome in the initiation of spontaneous uveoretinitis in the *Aire^GW^*^/+^*Lyn^-/-^* mice or in the P2.U2^+/-^ TCR transgenic mice has not emerged from antibiotic depletion experiments (18, 21). These mice received a broad-spectrum antibiotic cocktail treatment from birth through 12 weeks of age, followed by funduscopic evaluation. Colony-forming unit counts from colonic contents confirmed effective depletion of both aerobic and anaerobic bacteria; however, uveitis incidence remained unchanged compared with untreated controls (18). It should be noted that a viral trigger is not ruled out by these experiments and it is also possible that a microbe that was resistant or partially resistant to the antibiotic depletion protocol was responsible. Future experiments could address this issue further by examining the P2.U2 TCR transgenic T cells for possible activation in gut tissues or other barrier locations and by use of germ-free mice.

## Role of lymphopenia in the spontaneous development of uveoretinitis in TCR transgenic mice and in mice with Aire deficiency

In a number of mouse models of autoimmune disease, the mice exhibit reduced numbers of T cells (lymphopenia) and this lymphopenia has been implicated in the development of disease (40, 41). Indeed, autoimmunity is a common occurrence in Omenn’s syndrome in human, which is caused by genetic partial loss of function of the VDJ recombination machinery, and hence a decreased output of B cells and T cells (42).

In the uveoretinitis models discussed above, lymphopenia was implicated in uveoretinitis in both the β-galactosidase system and the HEL system. In the β-galactosidase neo-self-antigen system described above, the double transgenic mice are highly resistant to spontaneously developing uveoretinitis (24). The only manipulation that resulted in uveoretinitis was to adoptively transfer the anti-β-galactosidase TCR transgenic T cells into lymphocyte deficient (i.e., lymphopenic) neo-self-antigen single transgenic mice, and additionally to deplete CD25^+^ cells (including Tregs) from the adoptively transferred TCR transgenic T cells. Thus, lymphopenia contributed to the development of uveoretinitis in this model, but disease also required a second defect in immune tolerance, namely a reduction in Tregs. In the HEL/anti-HEL double transgenic mice, severe uveoretinitis developed spontaneously starting on day 20–21 after birth, a time at which these mice had marked lymphopenia (23). Naive anti-HEL TCR transgenic T cells derived from mice not expressing HEL could induce uveoretinitis upon adoptive transfer into the HEL neo-self antigen single transgenic mice with normal endogenous T cells, but this required additionally infection with a virus expressing the HEL epitope (25), therefore the anti-HEL TCR transgenic T cells were pathogenic in mice without lymphopenia, but only following a viral infection with engineered molecular mimicry.

The spontaneous uveoretinitis observed in *Aire^-/-^* mice and in *Aire^GW/+^Lyn^-/-^* mice likely does not involve lymphopenia, as defects in Aire decrease negative selection and thus would not be expected to result in lymphopenia beyond what occurs normally in mice after birth. The possibility of lymphopenia in the P2.U2^+/-^ transgenic mice was not explicitly addressed in their initial characterization. Given that when these transgenic T cells were generated in *IRBP^-/-^* mice, only 12% of CD4 T cells expressed an IRBP P2-specific TCR, it appears that this TCR transgenic has inefficient expression of one or both of the TCRα and TCRβ-expressing transgenes and allows generation of many cells with endogenously generated TCRs. Thus, the detectably increased level of negative selection seen in these mice would likely at most cause a very mild lymphopenia.

## Mechanism of spontaneous autoimmune uveoretinitis in *Aire^GW/+^Lyn^-/-^* mice

A key question in each of the spontaneous mouse models of uveitis is how do retina-specific CD4 T cells become activated to a potentially pathogenic effector phenotype. As mentioned above, in the case of the R161H and R161M IRBP-specific TCR transgenic mice, molecular mimicry with a gut microbe appears to be necessary to prime some of the R161 TCR expressing T cells to achieve a pathogenic effector phenotype. In the case of the HEL/anti-HEL double transgenic system and the adoptive transfer model of β-galactosidase specific T cells into β-galactosidase neo-self antigen transgenic mice, lymphopenia is an important contributor to the development of a pathogenic T cell response. In the absence of lymphopenia, adoptively transferred anti-HEL TCR transgenic T cells can induce eye inflammation if the neo-self antigen transgenic mice are infected with a virus with engineered molecular mimicry. In the case of the P2.U2 IRBP-specific TCR transgenic T cells, they are clearly capable of inducing substantial retinal inflammation and tissue damage, but evidence for a role of the microbiota is currently lacking and lymphopenia is unlikely. Thus, future studies will be required to provide insight into the mechanism by which these T cells acquire a pathogenic phenotype.

In both *Aire^-/-^* mice and *Aire^GW/+^ Lyn^-/-^* mice, IRBP has been shown to be a critical self antigen for the retinal inflammation and tissue damage that develops spontaneously in a substantial fraction of the mice (21, 43). Therefore how IRBP-specific T cells initiate autoimmunity in these mice is a key question. Aire drives expression of IRBP in the thymus and this expression promotes negative selection and to a lesser extent Treg lineage commitment of developing T cells recognizing IRBP epitopes presented by class II MHC molecules. Therefore, in these mice, IRBP-specific CD4 T cells can develop without undergoing negative selection and join the peripheral T cell population. The *Aire^GW/+^* mice express a small amount of residual IRBP in the thymus (10), so it is likely that these mice have fewer IRBP-specific CD4 T cells that evade negative selection and/or a small number of IRBP-specific CD4 T cells that become Treg in the thymus. In addition, it is possible that the highest affinity IRBP-specific CD4 T cells undergo apoptosis in the *Aire^GW/+^* mice but not in the *Aire^-/-^* mice (44). One or another of these effects on IRBP-specific CD4 T cell populations may be responsible for the lower incidence of uveoretinitis in the *Aire^GW/+^* mice compared to *Aire^-/-^* mice.

In addition, Aire has been shown to play a role in peripheral tolerance of T cells through effects on antigen presenting cells in peripheral lymphoid organs (45), whether or not a compromise in this function of Aire contributes to uveoretinitis in *Aire^GW/+^ Lyn^-/-^* mice has not been addressed.

## Deletion of Lyn enhances antigen presentation of IRBP P2 by dendritic cells

For the spontaneous uveoretinits in the *Aire^GW/+^ Lyn^-/-^* mice, one important question is what is the role of Lyn-deficiency in disease initiation. To identify the cellular mediator of uveoretinitis with regard to the Lyn deficiency, *Aire^GW/+^* mice carrying two floxed *Lyn* alleles were bred to include cell type–specific Cre-recombinase lines targeting dendritic cells (DCs; *Cd11c-Cre*), macrophages and neutrophils (*LysM-Cre*), or B cells (*Mb1-Cre*). Cohorts underwent funduscopic screening from 6 weeks through 6 months of age. Strikingly, only DC-specific *Lyn* deletion replicated the uveoretinitis phenotype, affecting 13 of 33 *Aire^GW/+^Cd11c-Cre Lyn^fl/fl^* mice (Aire^GW/+^ DC-*Lyn^-/-^*), while mice with macrophage/neutrophil or B cell–targeted deletion of Lyn failed to develop disease (18).

The uveoretinitis in *Aire^GW/+^* DC-*Lyn^-/-^* mice recapitulated key features of the global Lyn-deficient model: early onset preceding systemic autoimmunity, evidence of an autoimmune response to IRBP, and retinal infiltration by activated T cells, including those specific for an IRBP CD4 T cell epitope, as detected by class II MHC tetramers (P2/I-A^b^ tetramers) (18). CD11c-Cre is known to induce deletion in over 95% of conventional DCs and in a lower frequency in plasmacytoid DCs (46). These results suggest that, in the context of *Aire^GW/+^*, *Lyn* deficiency restricted to DCs is sufficient to trigger autoimmune uveoretinitis, despite Lyn’s expression across multiple immune lineages and its established role in B cell–mediated autoimmunity in other contexts. Of course, with any cell type deletion experiment using Cre/lox, there may be some deletion in additional cell types, so this is a caveat for this conclusion. When CD11c-Cre was combined with a flox-based genetic reporter, it was found to induce reporter expression not only in conventional DCs but also in some tissue macrophage populations, such as alveolar macrophages (47).

The hypothesis that the deletion of *Lyn* primarily promoted uveoretinitis by its effects on DCs is supported by another observation. DCs from eye-draining and non-eye draining lymph nodes of wild type and *Lyn^-/-^* mice were highly enriched with anti-CD11c-magnetic beads and the resulting populations were tested for their ability to stimulate a T cell hybridoma that recognizes the IRBP P2 epitope. It was found that only antigen-presenting cells from eye-draining lymph nodes were stimulatory and moreover that the enriched DCs from *Lyn^-/-^* eye-draining lymph nodes were several-fold more stimulatory than the enriched DCs from the corresponding lymph nodes of C57BL/6 mice (18). Taken together, these two types of experiments make it likely that the major effect of *Lyn* deletion was manifested by conventional DCs by increasing their ability to present endogenous self antigen to autoreactive CD4 T cells.

Why *Lyn*-deficiency enhances antigen presentation of IRBP by the DCs in the eye-draining lymph nodes is not clear at this time. The T cell hybridoma that was used to assess antigen presentation of IRBP is not dependent upon CD28 costimulation, so this observation most likely reflects more P2/I-A^b^ ligands on the surface of *Lyn^-/-^* DCs. While the retina is not richly endowed with dendritic cells as is seen in barrier tissues, dendritic cells have been observed in some anatomical locations in the eye (48), so it is possible that IRBP is picked up in the eye and taken to the draining lymph nodes by DC trafficking via the lymphatics. In this scenario, Lyn-deficiency could affect uptake or trafficking. Alternatively, release of IRBP from retinal neurons, either as free protein or in some sort of subcellular vesicle, could travel via lymph fluid to the lymph node and be taken up there with increased efficiency by Lyn-deficient DCs. A recent study using the R161 anti-IRBP TCR transgenic model of spontaneous uveitis has found that selective depletion of ocular DCs did not prevent uveoretinitis, whereas systemic depletion did (23), perhaps favoring the latter model. In either case, Lyn-deficiency could also make the antigen-presenting DCs better able to stimulate an effector CD4 T cell response, an issue which has not been addressed by the experiments to date.

## Characterization of transcriptional patterns of P2-specific CD4 T cells in retina and lymph nodes of *Aire^GW/+^Lyn^-/-^* mice

To gain insights into the nature of the T cell autoimmune response in the *Aire^GW/+^Lyn^-/-^* mice, the phenotypes of IRBP P2-specific CD4 T cells in these mice were characterized by single cell transcriptomics (49). P2-specific CD4 T cells in the retinas of mice with early inflammation (6–7 weeks) were enriched with P2/I-A^b^ tetramers and analyzed by single-cell RNA sequencing (scRNA-seq). Individual cells were clustered by similarity (Seurat clustering and UMAP two-dimensional representation) and grouped into four clusters, identified as: 1) a Th1 effector population expressing Tbx21 (encoding Tbet), IFN-γ, Runx3 (a Tfh program repressor) and trafficking molecules Selplg (PSGL-1) and CXCR6; 2) a putative T regulatory 1 (Tr1) population (50) expressing c-Maf, IL-10, and high levels of inhibitory receptors LAG3, PD-1, Tigit, and CTLA4, alongside anergy-associated transcripts Nt5e and Izumo1r; 3) a FoxP3+ Treg population; and 4) a proliferating CD4 T cell population, expressing Mki67 and other proliferation-associated transcripts. Pathway enrichment of each cluster’s top 50 differentially expressed genes corroborated these identities. Collectively, these findings suggest that uveoretinitis in *Aire^GW^*^/+^*Lyn^-/-^* mice is driven primarily by Th1 inflammation targeting IRBP, accompanied by a substantial Tr1-like anti-inflammatory response that, despite comprising the majority of autoantigen-specific CD4^+^ T cells, failed to prevent progressive retinal destruction (49). Interesting in this regard is a study from Raverdeau et al. (51), which found that retinoic acid, which is present in the retina, can contribute to development of Tr1 cells, which may explain the high frequency of Tr1-like IRBP-specific T cells seen in inflammed retinas of *Aire^GW/+^ Lyn^-/-^* mice. Interestingly, induced production of retinal self-antigen-directed Tr1 cells was protective against both IRBP peptide-induced uveoretinitis (EAU) and the spontaneous uveoretinitis seen in the HEL transgenic/anti-HEL TCR transgenic mice (51).

Recently, a single cell transcriptomic analysis of autoreactive CD4 T cells infiltrating the pancreas of NOD mice with diabetes has been reported (52). These authors analyzed autoreactive CD4 T cells recognizing four different diabetes-related specificities, and interestingly, found that the transcriptional phenotypes of the autoreactive T cells were dependent to a considerable degree upon their specificity. Two different insulin-specific specificities (related to insulin B chain 9-23) were examined along with two different “hybrid” peptide epitopes. The latter are created by catalytic fusion of two different peptides that are generated in the chromaffin granules of pancreatic β cells, and hence are not present in the thymus, in contrast to insulin, which is expressed in the thymus under the control of Aire (44). Interestingly, CD4 T cells recognizing one of the hybrid peptides (a chromogranin A-insulin C fusion peptide implicated in diabetes onset) were dominated by effector type cells, which were present to a lesser degree in CD4 T cells recognizing the other hybrid peptide (insulin C-islet amyloid polypeptide), whereas CD4 T cells recognizing the insulin-derived epitopes were dominated by Treg and anergic T cell phenotypes. Thus, the T cells that would not be subjected to Aire-mediated central tolerance were most similar to the IRBP P2-specific T cells found in retinas undergoing autoimmune attack in *Aire^GW/+^Lyn^-/-^* mice. These results suggest that the lack of central tolerance to pancreatic β cell hybrid peptides may be a predisposing factor for development of diabetes in NOD mice and possibly in humans that develop type 1 diabetes. With regard to the spontaneous uveoretinitis seen in *Aire^GW/+^Lyn^-/-^* mice, it would be interesting to expand the transcriptomic analysis of IRBP specific CD4 T cells in the retinas in these mice with uveoretinitis to the other two known IRBP peptide epitopes in C57BL/6 mice to see to what extent their phenotypes differ.

Single cell transcriptomic analysis was also performed with P2 tetramer-binding CD4^+^ T cells isolated from eye-draining lymph nodes of *Aire^GW/+^Lyn^-/-^* mice with and without uveoretinitis, and also included P2-binding cells in the draining lymph node 5 days after immunizing *IRBP*^-/-^ mice with purified IRBP protein in complete Freund’s adjuvant. In this case, Seurat clustering was performed on cells combined from all three sample types, and seven distinct populations were visualized using UMAP. Each dataset contributed to all clusters, but there were substantial differences in cluster frequencies between the three samples. The seven clusters were identified as follows: 1) early activated T cells (expressing Ly6c1 and L-selectin/CD62L); 2) proliferating T cells (high expression of Mki67and other proliferation-associated genes); 3) transitional T cells (expressing lower Mki67 and adjacent to the proliferating cell cluster on the UMAP graph; these cells seemed to be exiting the cell cycle); 4) effector T cells (lacking Ly6c1 and L-selectin and expressing Th1 markers such as Tbx21 and IFN-γ and also expressing inhibitory receptors including LAG3); 5) putative memory T cells (expressing IL-7R and not expressing Ly6c1); 6) T cells with interferon response gene expression (expression of Ifit1 etc.); and 7) Tregs (FoxP3^+^). The cluster with an interferon-response signature was markedly increased in the uveoretinitis sample compared to the other two samples, suggesting that the Th1 effectors were actively secreting interferon-γ in the eye-draining lymph node in mice with an ongoing autoimmune response. The immunization sample was notable for having very few Tregs and more cells with transcriptional properties consistent with Tfh, compared to the two other samples. Putative memory and effector clusters shared expression of CXCR6 and CXCR3, one or both of which may be involved in trafficking of activated T cells to the eye. Differential gene expression analysis within T effector and putative memory populations across the three samples revealed a statistically significant increase in IL-2 receptor signaling-associated transcripts in uveoretinitis-derived Th1 effectors compared to disease-free counterparts (49). Thus, transcriptomic evidence suggests that CD4 T cells in the eye-draining lymph nodes of mice with uveoretinitis are secreting IFN-γ and IL-2 in greater amounts than in mice without disease, consistent with an active immune response. In addition, the presence of IRBP P2-specific CD4^+^ T cells of an early activated phenotype in both types of mice is consistent with the hypothesis that the autoimmune response is initiating in this location, rather than in gut-associated lymph nodes.

## Characterization of P2-specific CD4^+^ T cells in the eye-draining lymph nodes of individual *Aire^GW/+^Lyn^-/-^* mice

To overcome pooling limitations inherent in scRNA-seq analysis and to assess individual mouse variability, a flow cytometry approach was developed using a validated FoxP3^GFP^ reporter (23) introduced into *Aire^GW/+^Lyn^-/-^*mice. In the mice with the reporter, uveoretinitis incidence was 16/38, statistically indistinguishable from non-reporter controls (49). Combined with P2/I-A^b^ tetramer binding and surface staining for activation markers (Ly6C, CD62L, PD-1, LAG3, CXCR6), this strategy distinguished Tregs from early activated and effector populations, although interferon-signature, memory, and proliferating subsets identified by scRNA-seq were not explicitly resolved and likely were distributed among the LAG3-defined effector categories.

Mouse-to-mouse analysis of the eye-draining lymph node revealed that the mice without eye inflammation, assessed by funduscopy, had relatively few P2-specific CD4 T cells: between 3 and 21 such T cells, compared to between 41 and 714 cells in mice with uveoretinitis (49). In addition, there was a striking heterogeneity among the six disease-free animals analyzed. The mouse with the highest number of P2-specific CD4 T cells lacked Tregs and had mostly effector cells. Another mouse lacked Tregs and showed effector cell phenotypes among its six P2-specific cells. The remaining four mice harbored P2-specific FoxP3^+^ Tregs ranging from 14% to 66% of tetramer+ cells, with non-Treg populations predominantly comprising early-activated cells (Ly6C^+^ or Ly6C^−^CD62L^+^) in most of these mice. In the eye-draining lymph nodes of mice with uveoretinitis, the majority of P2-specific CD4^+^ T cells were in effector states that no longer expressed Ly6c1 or CD62L, and the fraction that were positive for the FoxP3-GFP reporter was very low (<5%). Collectively, these findings suggest that the mice with uveoretinitis had ongoing expansion of IRBP-specific CD4 T cells, and perhaps export to the eye. In addition, the examination of individual mice without eye inflammation indicated that they had a modest response to endogenous IRBP, which in some mice appeared to be restrained by Tregs, but in two mice was restrained by some other limitation, such as a small frequency of autoantigen-specific T cells and/or insufficient co-stimulation to permit a robust expansion of the cells (49), similarly to some of the adoptive transfer experiments with TCR transgenic T cells specific for retinal self antigen or neo-self antigen, discussed above.

The same flow cytometric analysis was then applied to a cohort of *Aire^GW/+^Lyn^-/-^FoxP3^GFP^* mice examined at 4–6 weeks of age, just prior to the onset of disease, and confirmed to lack detectable inflammation in the eye by funduscopy. These mice had, on average, approximately 30 P2^+^CD4^+^ T cells per eye-draining lymph node with substantial (~5-fold) inter-individual variation. Interestingly, the number of these autoantigen-specific CD4 T cells at 4–6 weeks was greater than that observed in the mice without uveoretinitis at 7–9 weeks of age. This result suggests that in the intervening time interval most of these cells had either died or left the eye-draining lymph nodes. As a control, age-matched *Lyn^-/-^FoxP3^GFP^* mice were analyzed and found to have negligible numbers of IRBP P2 tetramer-binding cells, possibly because Aire-mediated thymic deletion greatly reduced their numbers in the periphery. In addition, among pre-disease mice, there was a statistically significant inverse correlation between the total number of IRBP P2-specific CD4^+^ T cells and the fraction that were FoxP3^+^—mice with higher Treg proportions harbored fewer autoreactive cells (49).

The results from these flow cytometry experiments suggest that Treg frequency within the P2-specific T cell compartment, or possibly more generally in the IRBP-specific CD4^+^ T cell compartment, may be a critical determinant of which *Aire^GW/+^Lyn^-/-^* mice do or do not go on to develop autoimmune eye disease. That is, a high Treg representation within the P2-specific CD4^+^ T cell population appears to limit the proliferation of the corresponding autoreactive CD4^+^ T cells and may additionally reduce the differentiation of effector T cells into a pathology-inducing state. Conversely, if the number of autoreactive Tregs is low, the effectors may be able to expand enough to reach a critical threshold required to establish substantial inflammation in the retina. Additionally, the observation that the subset of *Aire^GW/+^Lyn^-/-^* mice that have not shown evidence of eye inflammation by 9 weeks of age did not develop disease subsequently suggests that there likely is an active peripheral tolerance mechanism preventing autoimmunity, as could be supplied by Tregs.

## Role of Tregs in restraining expansion of P2-specific CD4^+^ T cells in the eye-draining lymph nodes of *Aire^GW/+^Lyn^-/-^* mice

Based on these observations, Treg were transiently depleted from *Aire^GW/+^Lyn^-/-^*mice without detectable eye inflammation. This was done by introducing into the mice the *FoxP3^DTR^* allele, which enables selective Treg ablation via diphtheria toxin injection (53). When diphtheria toxin was injected into 6 week old *Aire^GW/+^Lyn^-/-^Foxp3^DTR^* mice that lacked detectable eye inflammation as determined by funduscopy, approximately 25% of these mice developed uveoretinitis within two weeks following transient Treg depletion, whereas control mice remained disease-free (49). Strikingly, nearly all *Aire^GW/+^Lyn^-/-^Foxp3^DTR^* mice—including those that failed to develop clinical disease—exhibited an approximately 15-fold expansion of P2^+^CD4^+^T cells in the eye-draining lymph nodes, as measured 14 days after Treg depletion. The three mice developing uveitis had average or above-average P2^+^ cell numbers; however, several mice with comparably elevated P2^+^ populations remained disease-free, indicating that numerical expansion alone was insufficient for pathogenesis (49).

These findings establish that FoxP3^+^ Tregs actively suppress P2-specific CD4^+^ T cell expansion in *Aire^GW/+^Lyn^-/-^* mice, and strongly suggest that antigen-presenting cells in the eye-draining lymph nodes are presenting the P2 epitope of IRBP prior to disease-induced retinal tissue damage, in agreement with the experiments with *in vitro* stimulation of a P2-specific T cell hybridoma, described above.

Previous studies that imaged CD4 T cells in lymph nodes also implicated Treg in restraining autoreactive CD4 T cell expansion and concluded that this restraint involved an IL-2-mediated negative feedback loop whereby the IL-2 produced by the autoreactive T cells promoted activity of Treg that then restrained the proliferative expansion of the autoreactive T cells (54). Analysis of IRBP P2-specific CD4 T cells in eye-draining lymph nodes of *Aire^GW/+^Lyn^-/-^* mice fit this hypothesis well and suggest that mice with a high effector/Treg ratio might be predisposed to expand the effector cells and go on to develop uveoretinitis.

As described above, Treg have been shown to be able to protect against uveoretinitis in a number of different circumstances, including in the β-galactosidase neo-self antigen situation (28, 29) and with R161 IRBP-specific TCR transgenic T cells injected into the eye microenvironment (31).

Critically, nearly all Treg-depleted *Aire^GW/+^Lyn^-/-^Foxp3^DTR^* mice exhibited expansion of P2^+^ CD4 T cells, but the majority did not develop uveoretinitis. This observation reveals that Treg-mediated suppression represents only one tier of peripheral tolerance and implies the existence of additional peripheral tolerance checkpoints that operate downstream of initial T cell priming and clonal expansion. Potential mechanisms include: tissue-resident tolerance barriers (e.g., ocular immune privilege factors, such as programed death-ligand expression, or an immunosuppressive microenvironment), insufficient effector differentiation into tissue-homing phenotypes despite numerical expansion, or Treg-independent tolerogenic pathways, such as clonal anergy, tolerogenic dendritic cells, or metabolic constraints (49).

## Spontaneous uveoretinitis of *Aire^GW/+^Lyn^-/-^* mice and human uveitis

Human non-infectious uveitis represents a heterogeneous spectrum of ocular inflammatory disorders that can either be part of an inflammatory disorder affecting multiple tissues, often including skin, joints, or intestines, or can be “standalone” uveitis which is limited to the eye (19). In either case, immunological mechanisms continue to be debated with regard to the relative contributions of adaptive autoimmunity, which may target retinal autoantigens including IRBP, and overly active innate inflammatory effects. Mouse models of uveitis are perhaps useful in illustrating basic principles that may relate to one or more types of human uveitis. In any case, whether or not the uveoretinitis that is seen in *Aire^GW/+^ Lyn^-/-^* mice is mechanistically related to one or another type of human uveitis is unknown. As mentioned above, our goal in creating these mice was to test whether two genetic defects that adversely affect distinct aspects of T cell tolerance might lead to synergistic susceptibility to the spontaneous development of organ-specific autoimmunity. The fact that uveoretinitis emerged in these mice presumably relates in part to the fact that central immune tolerance of CD4 T cells to IRBP is under the control of Aire. Why organs that express Aire-regulated TSAs and are subjected to autoimmunity in *Aire^-/-^* mice fail to be the subject of autoimmune attack in *Aire^GW/+^ Lyn^-/-^* mice is not known at this time.

## Single organ autoimmune disease vs. multi-organ autoimmunity

As the two mutations engineered into *Aire^GW/+^Lyn^-/-^* mice are believed to compromise general aspects of immune tolerance to a large number of self antigens expressed in one or a few organs (TSAs), it is surprising that the main organ-specific autoimmune disease seen in these mice is in a single organ in 50% of the mice and no other organ at a substantial frequency. This contrasts with the involvement of multiple organs seen in Aire^-/-^ mice and humans with APS-1 (9). Aire^GW/+^ mice have some residual Aire function and a small amount of residual expression of TSAs in the thymus, but why the eye is especially susceptible to severe organ-specific autoimmunity and other organs are not is a mystery at this time. One relevant factor is likely to be the influence of genetic background, since C57BL/6 is generally thought to be a relatively autoimmunity-resistant background. For example, whereas Aire^GW/+^ C57BL/6 mice have only a small amount of organ-specific inflammation in lachrymal and salivary glands, Aire^GW/+^ NOD mice have multi-organ autoimmunity, including inflammation of the thyroid gland and the sciatic nerve, in addition to uveoretinitis (10). Another well established example of a genetic mutation exhibiting different autoimmune disease on different genetic backgrounds has been reported for mutation in the T cell inhibitory receptor PD-1. Originally, Pdcd1^-/-^ mice were generated on the BALB/c background, and these mice were reported to exhibit inflammation of the heart muscle (cardiomyopathy) (55). Subsequently, this mutation was bred onto NOD mice with different class II MHC haplotypes, and now a variety of additional organs were subject to autoimmune disease (56). In any case, future studies to address why the eye autoimmunity is so prevalent in *Aire^GW/+^Lyn^-/-^* C57BL/6 mice may provide significant insights.

## Mechanisms by which *Aire^GW/+^Lyn^-/-^* mice spontaneously develop autoimmune uveoretinitis

Based on the studies described above, the following is a working model for how spontaneous uveoretinitis develops in 50% of *Aire^GW/+^ Lyn^-/-^* mice (**Figure 1**). The effect of partial deficiency in Aire is mainly due to its rule in medullary thymic epithelial cells (mTec’s), where Aire drives expression of tissue restricted autoantigens (TSAs), including IRBP. CD4^+^ single positive thymocytes that are specific for the P2 epitope of IRBP are subjected either to negative selection or to a lesser extent to differentation into Treg, which can exit to the periphery. In mice with defects in Aire, P2-specific CD4^+^ thymocytes with diverse avidities for this self peptide can mature and exit to the periphery, but they are generally limited in their ability to cause pathology. Indeed, there is only a 3% uveoretinitis incidence in *Aire^GW/+^* mice, whereas addition of Lyn deficiency increases disease incidence to 50%. Aire also has a documented role in peripheral T cell tolerance (45), and the extent to which the compromise in this role contributes to uveoretinitis in *Aire^GW/+^ Lyn^-/-^* mice has not been addressed. The working model for how Lyn deficiency increases the incidence of unveoretinitis is that it does so by making DCs in the eye-draining lymph nodes more able to activate P2 IRBP-specific CD4 T cells, which are present due to the defect in central tolerance to this autoantigen. Evidence in favor of this hypothesis includes use of CD11c-Cre to delete Lyn primarily (although not exclusively) in DCs and also experiments that show that Lyn-deficient DCs from eye-draining lymph nodes have more P2/I-A^b^ peptide complexes to present to P2-specific CD4 T cells than their wild type DC counterparts. How DCs in the eye-draining lymph nodes acquire IRBP to allow them to process it and present IRBP peptides is not entirely clear. DCs in the eye (48) may pick up IRBP there and then traffic to the draining lymph node or alternatively either free or microvesicle-associated IRBP may travel via lymphatic drainage to the lymph node where resident DCs could internalize it. It is possible and perhaps likely that Lyn-deficient DCs are also better able to prime a pathogenic effector T cell response in other ways beyond just having more P2/I-A^b^ to present. Effects of Lyn-deficiency on DC functions are thought to reflect the ability of Lyn to promote the function of a variety of inhibitory receptors in these cells (17), and therefore multiple properties of DCs may be affected by its absence.

**Figure 1:**
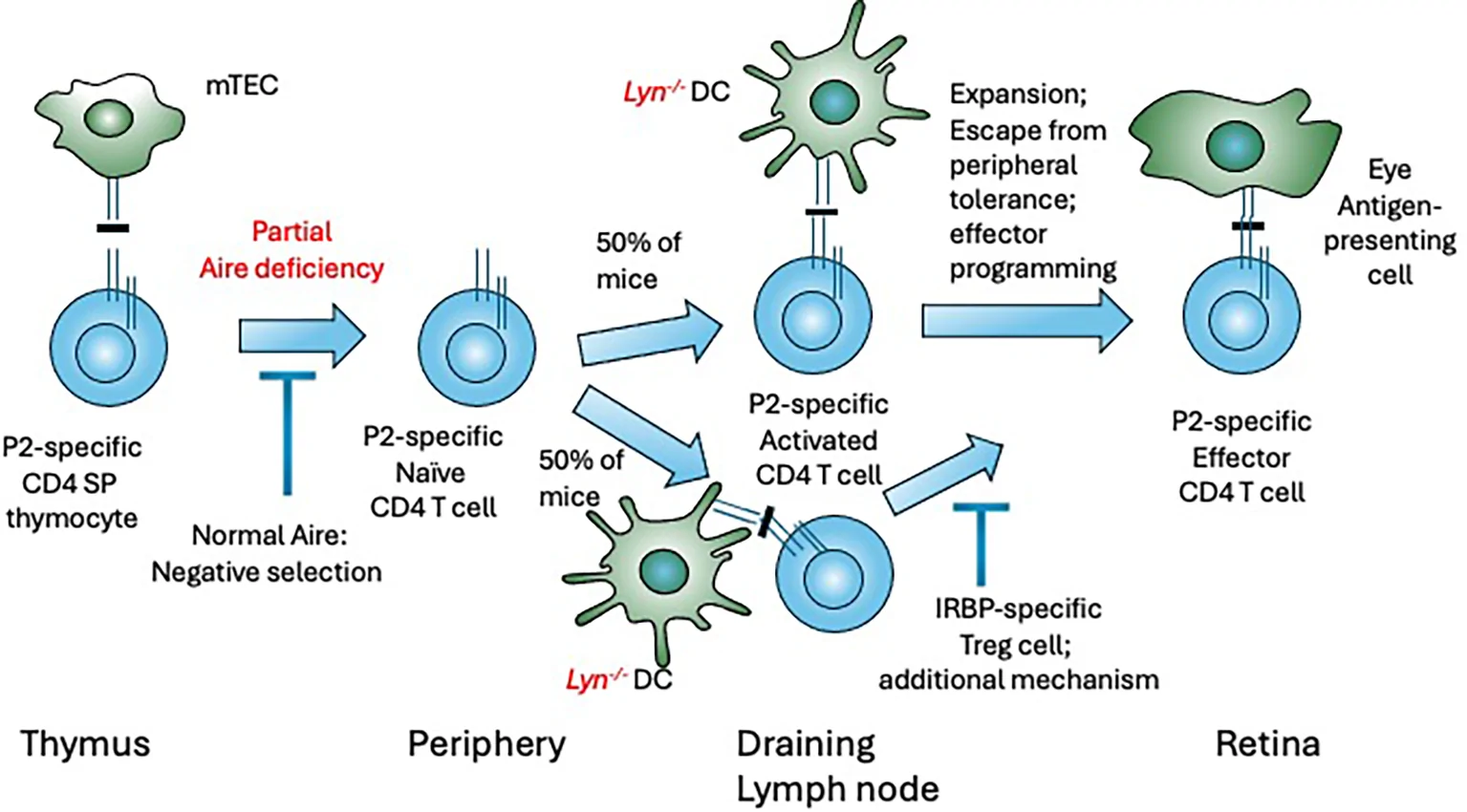
Working model for immunological mechanisms leading to uveoretinitis in 50% of *Aire^GW/+^ Lyn^-/-^*mice. Partial Aire deficiency leads to greatly reduced IRBP expression in medullary thymic epithelial cells (mTEC), and hence decreased presentation, leading to decreased negative selection of IRBP P2-specific thymocytes. Therefore, there are a greater number of naive P2-specific CD4 T cells in the periphery of mice with partial or complete defects in Aire. Lyn-deficiency is proposed to act primarily by increasing the ability of dendritic cells (DCs) to present antigen and activate autoreactive T cells. This appears to take place in the eye-draining lymph nodes. In 50% of mice, the P2-specific CD4 T cells proliferate and are programed to be Th1 effectors that can traffic to the retina and, upon reacting to P2/MHC class II complexes in that location, can initiate a destructive inflammation. Lyn-deficiency may also increase activation at this stage. For example, Lyn-deficient DCs have been shown to have enhanced cytokine production following TLR stimulation (17, 57). In the 50% of *Aire^GW/+^ Lyn^-/-^*mice that do not develop uveoretinitis, there appear to be at least two mechanisms limiting pathogenesis. In some mice, Treg (presumably recognizing retinal autoantigens) inhibit clonal expansion and possibly also effector differentiation of the naïve P2-specific CD4 T cells. Transient deletion of Tregs leads to uveoretinitis in some but not all of the mice, implying a Treg-independent mechanism of peripheral tolerance also acting in the eye-draining lymph node. An additional autoimmune disease checkpoint acting in the retina cannot be ruled out at this time. See text for additional details.

Activation and expansion of the P2-specific CD4^+^ T cells is observed in the eye-draining lymph nodes of the *Aire^GW/+^ Lyn^-/-^* mice before and during the critical period of disease susceptibility (6–9 weeks of age), and both clonal expansion and effector differentiation of P2-specific CD4 T cells are stronger in mice with uveoretinitis, as observed by funduscopy, compared to mice without detectable eye inflammation. Clonal expansion of P2-specific CD4 T cells in most mice without disease is restrained by Treg, most likely Treg recognizing retinal autoantigens. A minority of mice may have small numbers of P2-specific CD4 T cells in the draining lymph node due to stochastic generation of few such cells in the thymus (49), although study of more mice would be needed to clearly establish this point. Whereas all mice without uveoretinitis at 9 weeks of age exhibit clonal expansion of P2-specific CD4 T cells following transient depletion of Treg, only a minority of these mice go on to develop uveoretinitis within the next 14 days. Therefore, we hypothesize that in the mice without uveoretinitis, Treg are only one mechanism preventing disease and that a second peripheral tolerance mechanism is also operating, although the nature of this second mechanism is not yet evident. One limitation of the studies to date analyzing P2-specific CD4 T cells and their phenotypes in the eye-draining lymph nodes is that some of these T cells had likely exited the lymph node and potentially trafficked to retinal tissue. Therefore, future studies should also employ techniques to prevent exit of activated T cells from lymph nodes, as was done in a study of autoreactive T cells of another specificity (54). In the mice that develop disease, in addition to clonal expansion of the P2-specific CD4^+^ T cells in the eye-draining lymph nodes, these cells show Th1 effector differentiation and acquisition of chemokine receptors that may promote entry into the retina. P2-specific effector Th1 cells are found in the eye tissue of these mice, along with many P2-specific T cells with a transcriptional signal consistent with their being Tr1 anti-inflammatory cells. This possible anti-inflammatory mechanism is unable to prevent tissue damage in this model.

In mice, there are relatively few spontaneous models of organ-specific autoimmune disease with an endogenous TCR repertoire, and the *Aire^GW/+^Lyn^-/-^* mouse model provides a relatively new such model with some attractive properties. These include its defined genetic susceptibility loci with reasonably well defined effects on T cell tolerance mechanisms, a defined critical autoantigen (IRBP), a discrete window of disease incidence, incomplete penetrance which enables correlation of hypothesized parameters of relevance with disease initiation, and the ability to detect early autoimmune inflammation in a non-invasive way (funduscopy). Indeed, the identification of IRBP as the critical autoantigen in these mice has enabled precise tracking and comprehensive analysis of autoreactive cells throughout disease evolution (49), although further studies are clearly warranted to provide additional evidence for hypothesized immunological mechanisms.

Studies to date have suggested that a key checkpoint differentiating the mice that develop uveoretinitis from those that do not is present in the eye-draining lymph node. This is another working hypothesis, which deserves additional study, especially given studies indicating that the eye is a tissue that is particularly immunosuppressive and has specialized vasculature that may impede entry of T cells (the blood-retina barrier) (28, 30, 33). A careful immunohistochemical analysis of retinas from mice without funduscopic evidence for retinal inflammation at 9 weeks of age would address whether or not there is a small degree of T cell infiltration at this point, which, if present, would suggest that an autoimmune disease checkpoint might also occur in the target tissue, similarly to what is seen in NOD mice. In the NOD mouse, the critical checkpoint that must be passed to enable disease progression appears to occur within the endocrine pancreas tissue, as peri-insulitis is seen in all individuals at a young age, including those that do not progress to diabetes (12, 13). In contrast, in *Aire^GW/+^Lyn^-/-^* mice, we have not detected eye inflammation in the mice that do not develop disease, whereas in mice with detectable eye inflammation, the tissue inflammation typically is progressive, leading to severe damage to the retinas of both eyes. Of course, there is likely to be initial priming of naïve CD4 T cells to islet β-cell autoantigens (insulin epitopes and hybrid peptide epitopes) in the pancreatic lymph nodes, so it should be kept in mind that type 1 diabetes in NOD mice likely involves an autoimmune checkpoint in the organ-draining lymph node as well as a checkpoint in the target tissue. Nonetheless, the relative importance of a lymph node checkpoint vs. a target tissue checkpoint for determining disease vs. lack of disease may differ between NOD mice and *Aire^GW/+^ Lyn^-/-^* mice. For this reason, *Aire^GW/+^ Lyn^-/-^* mice may be able to provide unique insights into the nature of disease initiation for organ-specific autoimmune disease.
